# Investigation of the motion of fullerene-wheeled nano-machines on thermally activated curved gold substrates

**DOI:** 10.1038/s41598-022-22517-1

**Published:** 2022-10-29

**Authors:** Mohammad Ali Bakhtiari, Saeed Seifi, Mahdi Tohidloo, Amir Shamloo

**Affiliations:** School of Mechanical Engineering, Sharif University of Technology, Azadi Ave., Tehran, Iran

**Keywords:** Biophysics, Computational biophysics, Molecular biophysics, Nanoscale biophysics

## Abstract

The current study presents one of the first investigations in which the simultaneous effect of the curved gold substrates and temperature changes on C_60_ and C_60_-wheeled nano-machines’ migration was evaluated. For this aim, the cylindrical and concave substrates with different radii were chosen to attain the size of the most appropriate substrate for nano-machines. Results indicated that the chassis' flexibility substantially affected the nanocar's mobility. Nano-machines' deviation from their desired direction was adequately restricted due to selected substrate geometries (The cylindrical and concave). Besides, for the first time, the effect of the substrate radius changes on nano-machine's motion has been investigated. Our findings revealed that adjusting the value of radius results in a long-range movement for nano-machines as well as a sufficient amount of diffusion coefficient even at low temperatures ($75\; \text{K}$ or $150\; \text{K}$). As a result, the aforementioned substrates could be utilized as the optimized geometries for C_60_ and nanocar at all temperatures. At the same time, the nanotruck displayed an appropriate performance merely on the small cylindrical substrate ($radius=17.5 \; \text{\AA}$) at high temperatures ($500\; \text{K}$ and $600\; \text{K}$).

## Introduction

In recent years, with the dramatic development in nanotechnology, some researchers have shown strong attention to the manipulation of nano-scale materials. Numerous theoretical studies and experimental researches have been accomplished in association with these materials^1–4^. A great number of methods are present for manipulating one molecule or a cluster of atoms on a surface^5^. However, there are some drawbacks to these methods contrasting with the performance of natural nano-manipulators^6,7^. First and foremost, they are not capable of working on abundant particles simultaneously^8^, and also, they are several orders of magnitude greater than the manipulated payloads^9^. Therefore, inspired by the transportation of atoms and molecules in nature in which molecules are in the same order of magnitude (or even smaller), investigations have attempted to fabricate manipulators whose sizes are in the same order of the corresponding payloads^9–11^.

Tour et al. succeeded in fabricating several molecular motors and machines, aiming to carry other nano-scale materials^9,12,13^. Due to the resemblance between these molecular motors and real cars, researchers named these synthesized molecular machines as nanocars, nanotrucks, or similar names^8,9,12,14,15^. In the prior models of nano-machines, three or four-wheeled nano-machines in which C_60_ was utilized as a wheel were fabricated significantly^12,16,17^. C_60_ is a well-known molecule that quite a few experimental and computational studies were reported its motion on different substrates^6,17,18^. Additionally, since all reported nanocars and nanotrucks employed with C_60_ fullerene wheels, it seems that the comparison between C_60_ and the nano-machines not only provides us with a better understanding of these machines, but may bring a breeding grounds for finding a correlation between them as well^19,20^. Within the following generation, p-carborane-based wheels were extended in multiple nanocars concerning chassis with different shapes and even more wheels^16^. Besides, the small size of these nano-machines enabled them to be utilized in a considerable number simultaneously, which qualified them for transporting payloads effectively.

It should be noted that before using these nano-machines, it is necessary to determine their class of motion using experimental or analytical methods^13^. Scanning Tunneling Microscopy (STM) is a practical measuring method that monitors numerous types of nanocars^21^. Shirai et al.^22^ and Zhang et al.^13^ are two primary groups who investigated the motion of the nanocars’ family using experimental studies. They analyzed the motion of several fullerene-wheel nanocars, at different temperatures, on a gold substrate. In addition, in some cases, they examined the effect of electric fields on nanocars’ motion. It can be observed that using STM, few images were captured in a minute, and the details of the motion were partially displayed^23^. In addition, STM suffers from several drawbacks, including being expensive and ridiculously time-consuming. Hence, it seems worthy to use computational simulation techniques, which are appropriate to measure the motion of these nano-structures in different conditions^24–27^.

Nevertheless, a few kinds of fullerene-wheel nano-machines have been analyzed via computational simulation techniques so far. Akimov et al.^28^ and Konyukhov et al.^29,30^ are two investigators who considered nanocars with rigid C_60_ wheels and rigid chassis. Although their simplifying assumptions allowed them to accomplish simulations more quickly, their model accuracy decreased drastically. In other words, by using rigid body molecular dynamics, the efficacy of chassis flexibility on the nanocar motion has been disassembled, and the chassis attachment to the substrate is not indicated properly. The issues mentioned above were solved by Nemati et al.^31^. They investigated the rotational and translational motions of nanocar and nanotruck on the gold substrate at different temperatures employing an all-atom model and classic atomistic dynamics to attain better accuracy. Their study helps us to better understand the thermally driven fullerene-based nano-machines with high controllability and maneuverability. Similarly, Mofidi et al. studied the motion of the C_60_-wheeled nano-machines on the graphene substrates at different conditions and temperatures^25^. They considered the diffusion coefficient of translational motion without taking into account the rotational motion, wheels, and chassis impacts on the mobility of nanocars. However, the motion of these nano-carriers on the curved shape substrate remains unexplored. For instance, gold nanotubes are recognized as outstanding nanomaterials with excellent load-carrying capacity and are malleable to different sizes and shapes^32^. Besides, In recent studies, gold nanotubes have been used as a promising candidate for multidisciplinary fields due to their specific features like large surface area, excellent adhesion properties, and corrosion resistance; some applications include electronic structure, biosensors, pharmaceuticals, and theranostic^32–35^.

The current study presents one of the first investigations in which the simultaneous effect of the curved gold substrate and temperature changes on nano-machine migration were explored. The cylindrical and concave substrates were chosen as the curved substrate due to their similar contacting surface to the nano-machine wheels. In addition, these substrates can simultaneously simulate the motion of nano-machines on the flat, downward, and upward step substrates. In the first step, the impacts of chassis' attachment on the motion of nano-machines were also studied. Eventually, a correlation between the motion of the wheels, nanotruck, and nanocar on different substrate sizes was obtained.

## Method

### Potential energy approach

Potential energy analysis is an outstanding tool, enabling us to predict the regime of motion of a nanotruck or nanocar on the variant substrate conditions.

**Figure 1:**
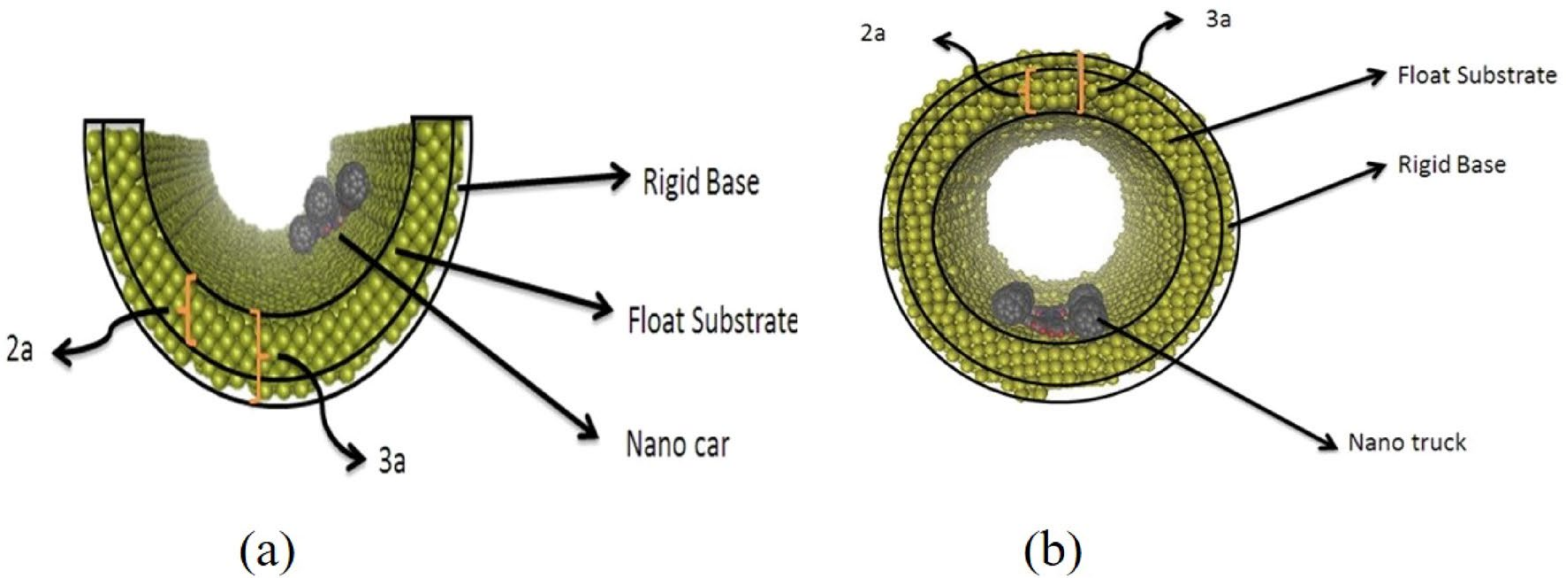
Description of simulation samples. (**a**) nanocar on the concave substrate and (**b**) nanotruck on the cylindrical substrate.

**Figure 2:**
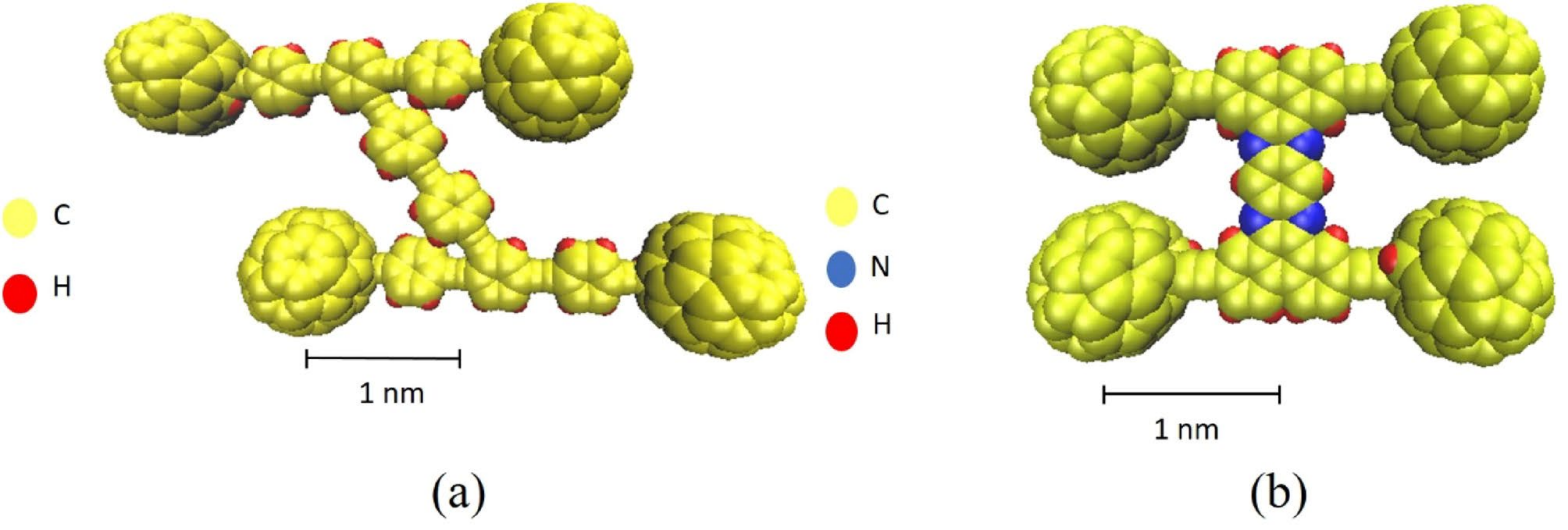
Top view of a fullerene-based molecular machine with four wheels: (**a**) Nanocar, the size of the exible-chassis machine is approximately $3  \; \text{nm} \times 4  \; \text{nm}$, (**b**) nanotruck, the chassis is practically rigid and due to the presence of nitrogen atoms in the chassis, it can potentially be attached to and carry other molecules. Its size is about $2  \; \text{nm} \times 3  \; \text{nm}$. In both molecules, the carbon, hydrogen, and nitrogen atoms are shown in gray, white, and blue, respectively.

**Figure 3:**
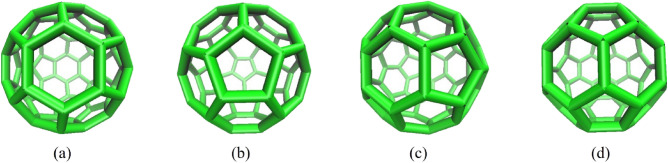
Orientations of C_60_ on gold surface (bottom view). (**a**) C_60_ on a hexagonal face (**b**) C_60_ on a pentagonal face (**c**) C_60_ on a bond between a pentagonal and a hexagonal faces (**d**) C_60_ on a bond between two adjacent hexagonal faces.

In this section, the potential energy of a C_60_, nanocar, and nanotruck on curve-shaped gold substrates, comprised of a floating substrate (inner layer) and a rigid base (outer layer), has been investigated (Fig. 1). In addition, Fig. 2 shows the schematic views of a $3 \; \text{nm} \times 4  \; \text{nm}$ flexible-chassis nanocar and $2  \; \text{nm} \times 3  \; \text{nm}$ stiff-chassised nanotrucks^9–12^. As Pishkenari et al. reported^24^, the orientation of ${C}_{60}$, nanocar, and nanotruck vigorously affected their potential energy on a gold substrate. They provided a comparison between four different C_60_ orientations, considering translational and rotational motion, and demonstrated that Hexa-down orientation has the most stable direction (Fig. 3).

### Simulation setup

In the current study, the motion of C_60_, nanocar, and nanotruck on the curved-shaped gold surfaces has been investigated using an atomistic dynamics approach. Simulations were performed at different temperatures in the range of $75\; \text{K}$ to $600\; \text{K}$, considering various radii ($17.5 \; \text{\AA}$ to $30 \; \text{\AA}$ for nonotruck and $20 \; \text{\AA}$ to $30 \; \text{\AA}$ for nanocar) on the curved surfaces. The minimum radius mentioned above, was considered for the geometries based on the size of C60, nanocar, and nanotruck. Thus, the substrates with $20 \; \text{\AA}$ and $17.5 \; \text{\AA}$ radii were selected for nanocar and nanotruck, respectively. Besides, the gold nanotube has been stable at high temperatures; as the previous studies are highlighted at $900\; \text{K}$ diffusion in the gold nanotube is intrusive, but atoms stick to the walls and do not move until $1200\; \text{K}$^33,34,36,37^.

To evaluate the efficacy of temperature and radii on the mobility of C_60_, the substrates' length was set to $27 a$, where $a$ stands for the gold lattice constant and assessed to be $4.078 \; \text{\AA}$^38^. The nanocar or nanotruck was posited on the inner layer of gold substrates, while the outer layer was considered rigid. The embedded atom method (EAM) potential, which extended based on density functional theory, is able to predict dislocation structures well analogous with experimental observations^39^. Thus, an EAM alloy potential, appropriate for modeling face-centered cubic (FCC) metals, and Molecular Mechanics (MM) force field was applied for modeling the interactions among the gold atoms and interaction within the nanotruck atoms, respectively^40,41^. Considering the harmonic style, bonds and angles are formulated as below:

1 $${E}_{bond} = {K}_{b} {(r-{r}_{0})}^{2}$$

2 $${E}_{angle} = {K}_{a} {(\theta -{\theta }_{0})}^{2}$$

In Eq. (1), ${K}_{b}$ represents the bond stiffness, and $r$ and ${r}_{0}$ stand for the bond distance and equilibrium bond distance, respectively. In addition, In Eq. (2), ${K}_{a}$ is the angle stiffness, and $\theta$ and ${\theta }_{0}$ are the angle and equilibrium angle, respectively^42^. Dihedral term style is presented in Eq. (3) as follows:

3 $${E}_{dihedral}=\frac{1}{2}{K}_{d1}\left(1+\mathrm{cos\varphi }\right)+\frac{1}{2}{K}_{d2}\left(1-\mathrm{cos}2\mathrm{\varphi }\right)+\frac{1}{2}{K}_{d3}\left(1+\mathrm{cos}3\mathrm{\varphi }\right)+\frac{1}{2}{K}_{d4}\left(1-\mathrm{cos}4\mathrm{\varphi }\right)$$

where $\mathrm{\varphi }$ is the dihedral angle and ${K}_{d1}$ to ${K}_{d4}$ represents the torsion stiffness parameters. The MM3 force field was performed to calculate the potential for these parameters, provided in Table 1, supposing that improper terms are negligible in the simulations^43–47^. Simulations were performed using a Large scale Atomic/Molecular Massively Parallel Simulator (LAMMPS) solver^48^, followed by visualizing the results on Visual Molecular Dynamics (VMD) software^49^.

**Table 1:** Parameters used in simulation of the nanocar and nanotruck.

Bonds parameters				
${K}_{b}(\frac{ev}{{ \text{\AA} }^{2}})$	${r}_{0}( \text{\AA} )$	Description		
$48.6652$	$1.212$	$C2 C2$		
$30.8837$	$1.313$	$C2 CA$		
$25.1593$	$1.392$	$CA CA$		
$14.3500$	$1.101$	$CA H$		
$34.5960$	$1.260$	$CA NA$		

Angles parameters				
${K}_{a}(\frac{ev}{{rad}^{2}})$	${\theta }_{0}$	Description		
$1.46619$	$\uppi$	$C2 C2 CA$		
$1.34141$	$2\uppi /3$	$C2 CA CA$		
$1.34141$	$2\uppi /3$	$CA CA CA$		
$1.12304$	$2\uppi /3$	$CA CA H$		
$1.34141$	$2\uppi /3$	$CA CA NA$		
$1.34141$	$0.638\uppi$	$CA NA CA$		

Dihedrals parameters				
${K}_{d1}(ev)$	${K}_{d2}(ev)$	${K}_{d3}(ev)$	${K}_{d4}(ev)$	Description
$0$	$0.000043$	$0$	$0$	$CA C2 C2 CA$
$0$	$0.000043$	$0$	$0$	$C2 C2 CA CA$
$0$	$0.650451$	$0$	$0$	$C2 CA CA CA$
$0$	$0.650451$	$0$	0	$C2 CA CA H$
$-0.0403$	$0.208144$	$0$	$0$	$CA CA CA NA$
$0$	$0.234379$	0.046	$0$	$CA CA CA H$
$0.0433$	$0.650451$	$0$	$0$	$CA CA CA NA$
$0$	$0.390271$	$0$	$0$	$H CA CA H$
$0$	$0.650451$	$0$	$0$	$H CA CA NA$
$0$	$0.433634$	$0$	$0$	$NA CA CA NA$
$0$	$0.433634$	$0$	$0$	$CA CA NA CA$

The substrate, the nanocar, and the nanotruck’s temperature was controlled using two Nose–Hoover thermostats. The first principle theorem, i.e. Restricted Hartree–Fock (RHF), was utilized for calculating charge distribution in the nanocar and nanotruck molecules. Calculations were obtained by employing the NWChem 6.5 package and $$6{-}31{G}^{**}$$ (d,p) basis set^50^. Despite the fact that there is little charge distribution in the nanocar and nanotruck, in the light that the gold substrate is electrically neutral, charge transfer between the nano-machines and gold substrate was neglected. Therefore, the electric field does not affect the motion of nano-machines on the gold substrate^51,52^.

**Figure 4:**
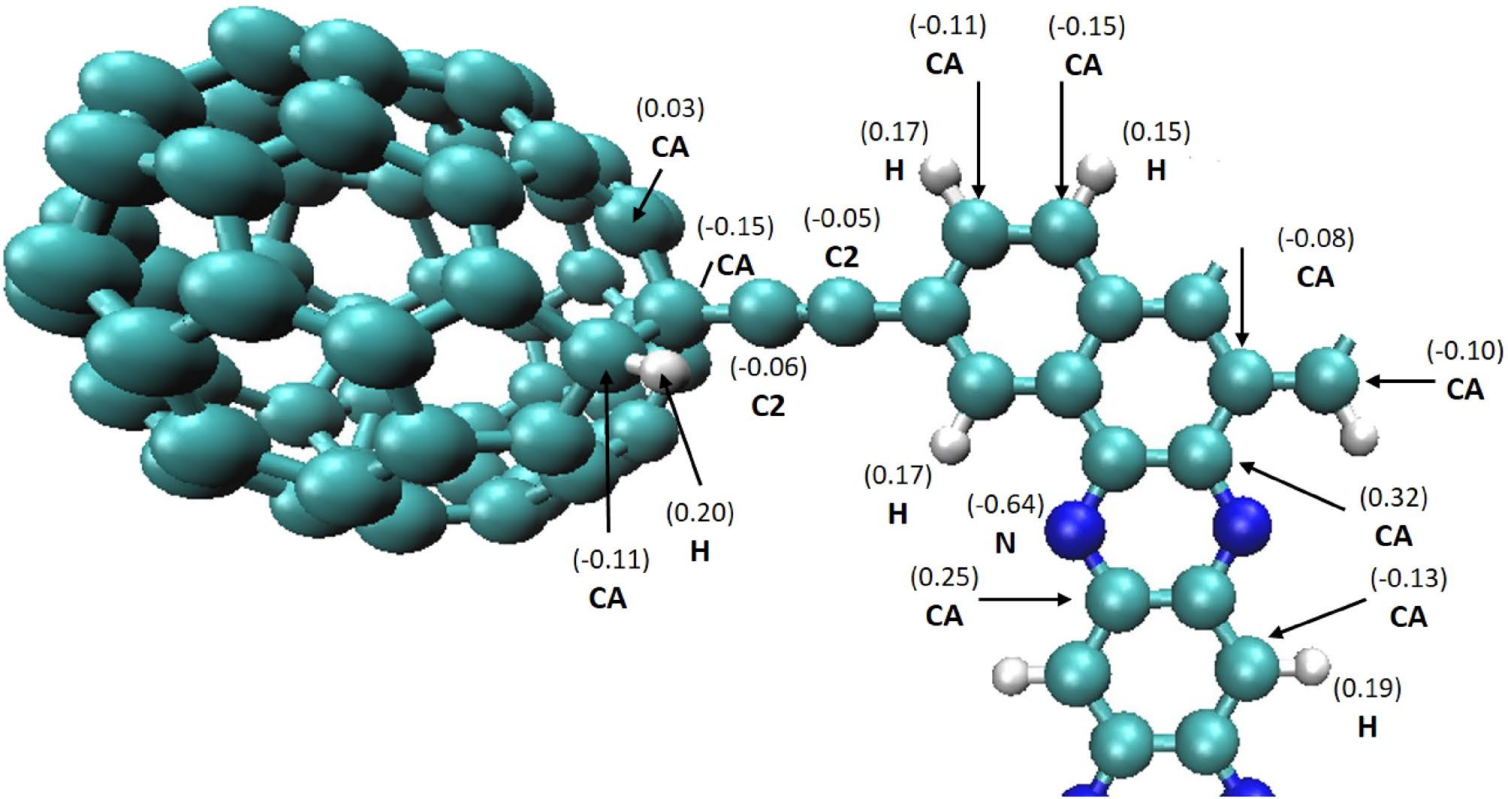
Different types of carbon atoms and charge distribution considered in the nanotruck.

In order to model the Van der Waals interactions between gold and carbon, hydrogen, and nitrogen atoms ($C{-}Au$, $H{-}Au$, and $N{-}Au$), Lennard–Jones 6–12 potential was utilized as:

4 $${E}_{Lj}=4\varepsilon \left[{\left(\frac{\sigma }{r}\right)}^{12}-{\left(\frac{\sigma }{r}\right)}^{6}\right]$$

where $\sigma$, $\varepsilon$, and r stand as potential parameters, representing the equilibrium distance, the well depth of the potential, and the distance between gold and carbon atoms at an equilibrium position, respectively. Table 2 summarizes the values for $\sigma$ and $\varepsilon$ parameters considering the same interaction parameters between gold and both types of carbon atoms (Fig. 4). Furthermore, the dimensions of the gold substrate in the horizontal and thickness orientations were considered large enough (three times the lattice constant), and the cut-off radius was set to ${r }_{cut{\text{-}}off}=13 \; \text{\AA}$ (${r }_{cut{\text{-}}off}>4 \sigma$)^53^.

**Table 2:** Parameters of the Lj potential.

$\varepsilon \; (ev)$	$\sigma \; (\text{\AA} )$	Description
$0.01273$	$2.994$	$C{-}Au$
$0.01315$	$2.611$	$H{-}Au$
$0.01423$	$2.886$	$N{-}Au$
$0.00190$	$3.460$	$C{-}C$
$0.00230$	$3.244$	$N{-}N$
$0.00204$	$2.673$	$H{-}H$
$0.00213$	$3.350$	$C{-}N$
$0.00197$	$3.647$	$C{-}H$
$0.00220$	$2.958$	$N{-}H$

## Results and discussion

### C_60_

**Figure 5:**
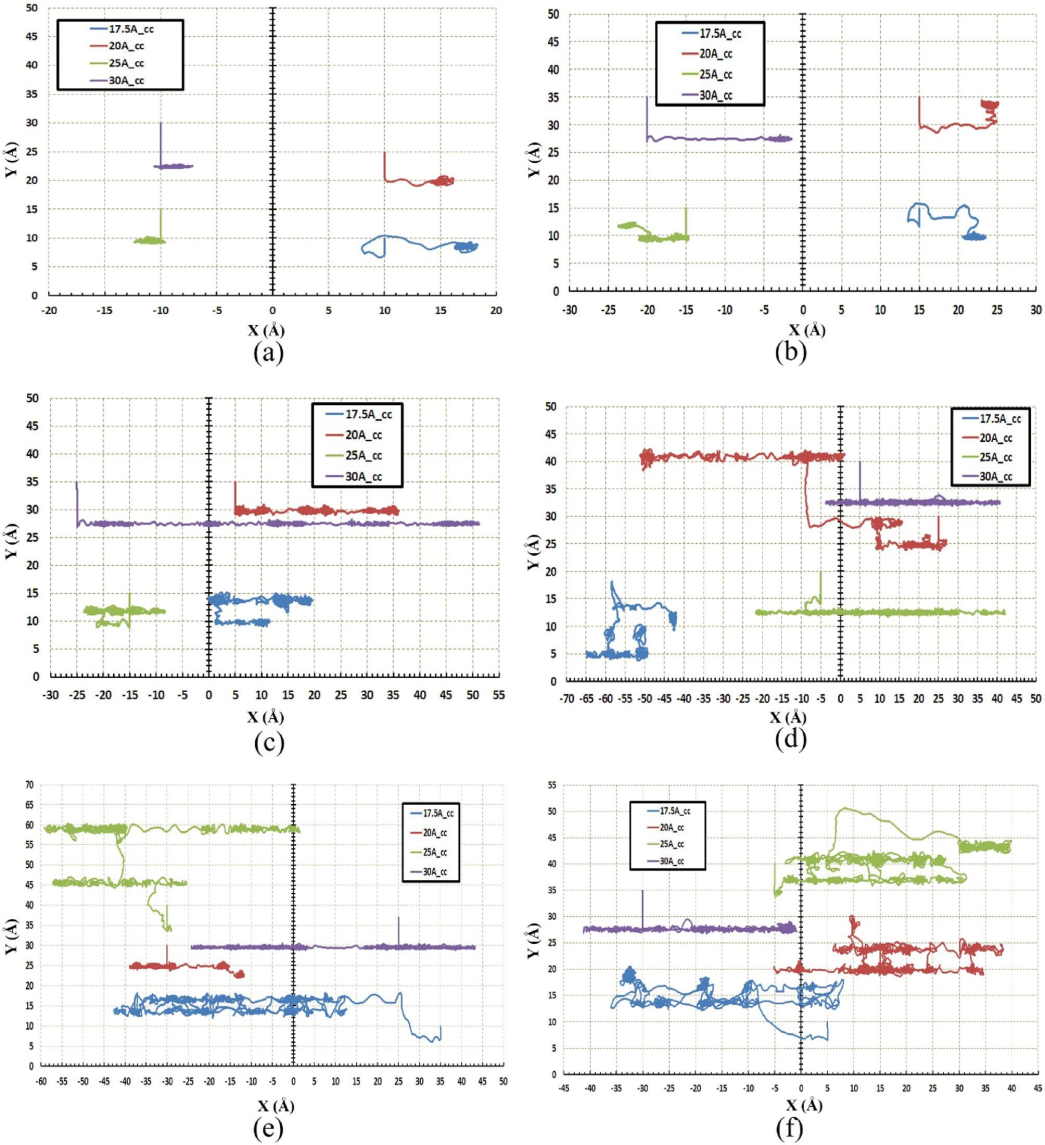
Trajectories of the C_60_ on the concave (cc) gold substrates during simulations (**a**) $75\; \text{K}$, (**b**) $150\; \text{K}$, (**c**) $300\; \text{K}$, (**d**) $400\; \text{K}$, (**e**) $500\; \text{K}$, and (**f**) $600\; \text{K}$. The initial deviation of the molecule at the beginning of the movement was because of adsorption towards the edges (due to the high surface-to-volume ratio).

**Figure 6:**
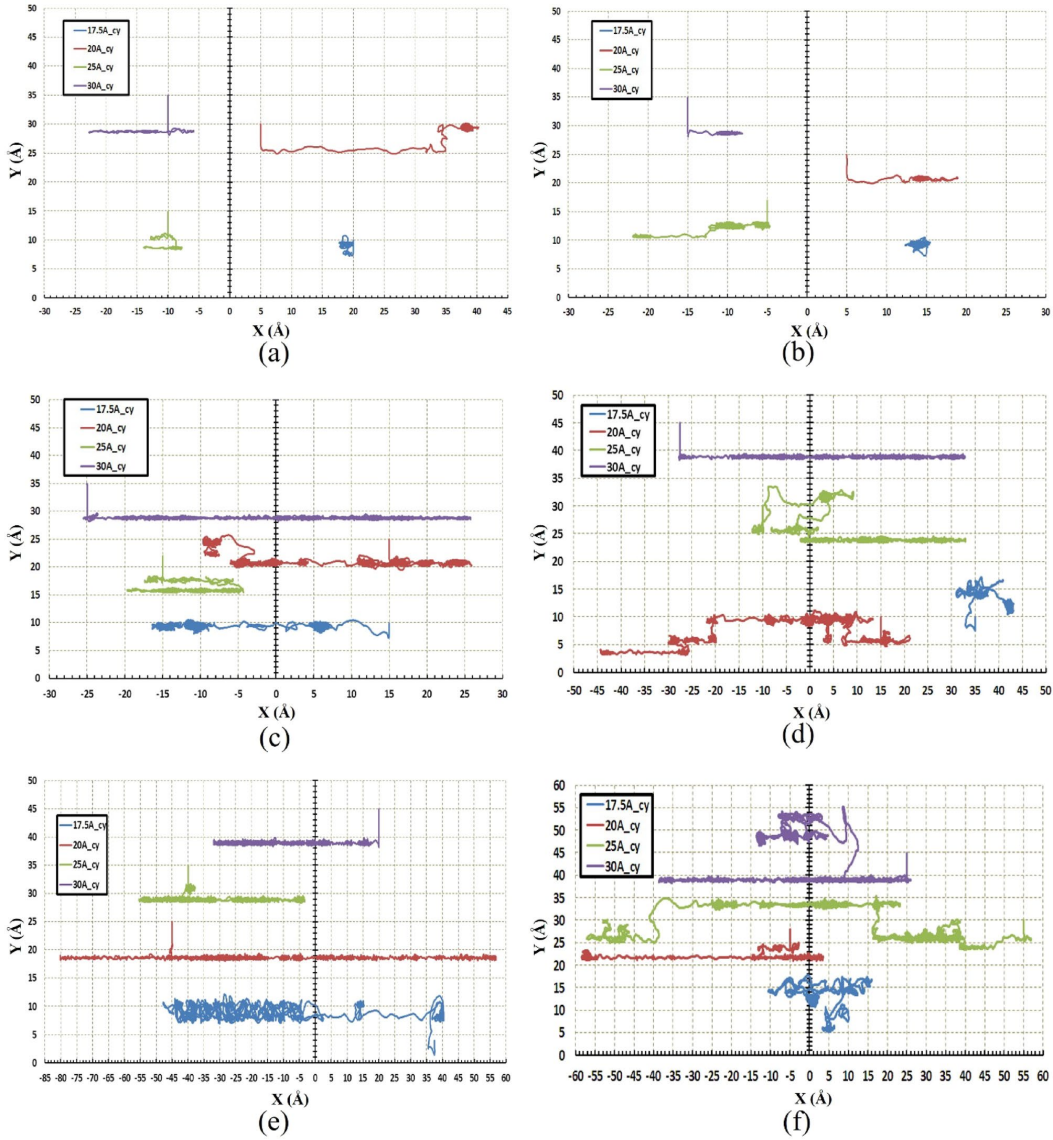
Trajectories of the C60 on the cylindrical (cy) gold substrates during simulations (**a**) $75\; \text{K}$, (**b**) $150\; \text{K}$, (**c**) $300\; \text{K}$, (**d**) $400\; \text{K}$, (**e**) $500\; \text{K}$, and (**f**) $600\; \text{K}$. At this geometry, there is no energetic point (the points with a high surface to volume ratio), so molecule movement deviation at cylindrical substrates is lower than the concave substrates.

**Figure7:**
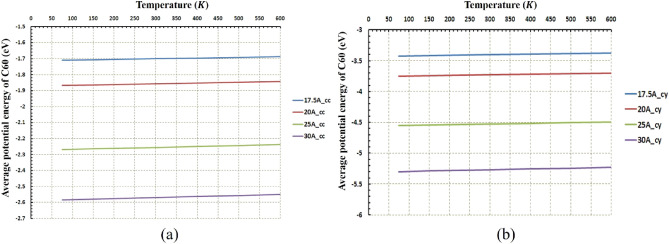
The average potential energy of C60 at different temperatures and radii (**a**) for the concave (cc) gold substrates and (**b**) for the cylindrical (cy) gold substrates. The temperature does not affect the average potential energy for both geometries. In cylindrical geometry, the molecule potential energy is double lower than the concave geometry.

**Figure 8:**
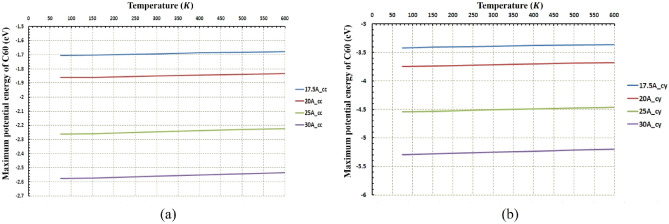
The Maximum potential energy of C60 at different temperatures and radii (**a**) for the concave (cc) gold substrates and (**b**) for the cylindrical (cy) gold substrates. The temperature does not affect the maximum potential energy for both geometries. In cylindrical geometry, the molecule's maximum energy is double lower than the concave geometry.

Figures 5 and 6 indicate the path of the C_60_ molecule on the gold substrates in cylindrical and concave geometries, respectively. The motion of the C_60_ molecule has less deviation from its direct pathway in the cylindrical geometry (Fig. 6). Likewise, this stability in motion was also observed in the concave-shaped substrates when the temperature and radii were increased (Fig. 5c–f). With increasing the radii, the number of gold molecules has increased, which decreases the substrate's surface-to-volume ratio. In other words, the surface effects were reduced, and as a result, the C_60_ molecule moved approximately in a direct path. Increasing the temperature separately, does not always lead to direct motion of C_60_ on substrates (e.g. Fig. 5c,f for $r=30 \; \text{\AA}$ and Fig. 6c,f for $r=30 \; \text{\AA}$). To express the cause of this issue and understand the parameters affected by temperature and radius, the potential energy between the C_60_ fullerene molecule and different gold substrates was investigated as well as changes in the diffusion coefficient (DC) of the C_60_ molecule. The average potential energy and maximum potential energy diagrams of the C_60_ molecule were represented in Figs. 7 and 8, respectively. As can be seen in both figures, the temperature changes over the mean and maximum potential energy at the same radii and geometry are almost negligible, which means that the adhesion between the C_60_ and the gold surface does not differ when the temperature increases at the same radii. Hence, this parameter is not a cause for less mobility of C_60_ in the aforementioned items (e.g., Fig. 5c,f for $r=30 \; \text{\AA}$, Fig. 6c,f for $r=30 \; \text{\AA}$). In addition, other results were obtained from Figs. 7 and 8, from which the average and maximum potential energy were increased, in the cylindrical substrate; it is twice as concave, with increasing radius in both geometries.

**Figure 9:**
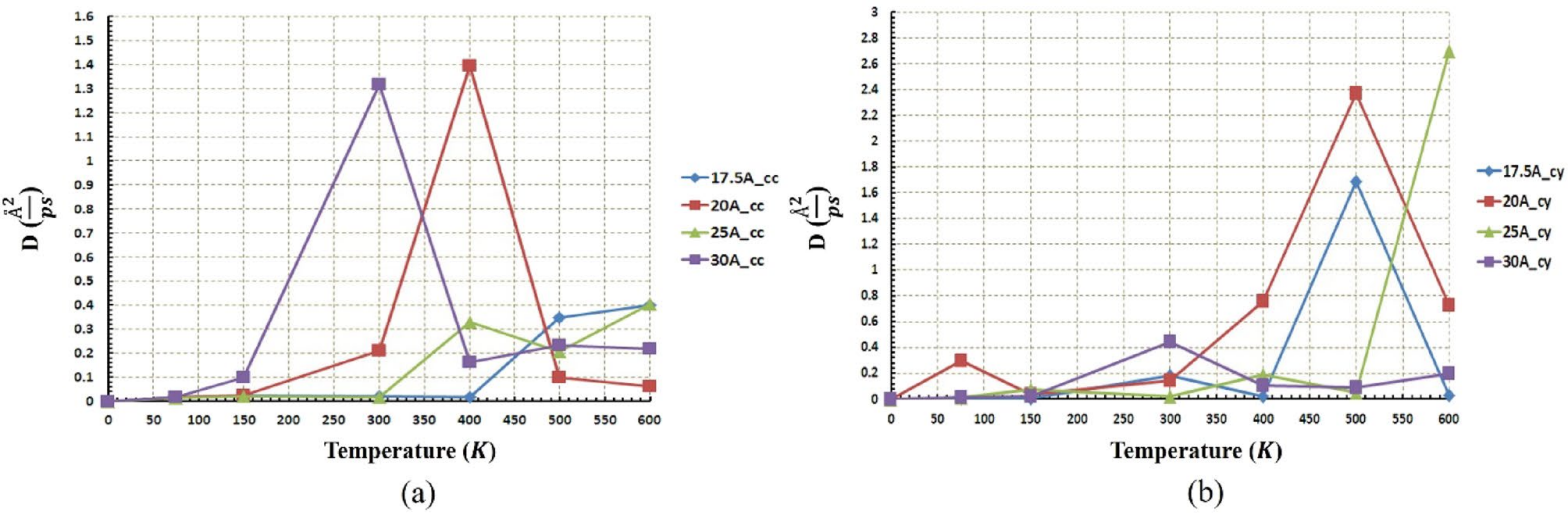
Diffusion coefficient of the C60 at different temperatures and radii (**a**) for the concave (cc) gold substrates and (**b**) for the cylindrical (cy) gold substrates. The diffusion coefficient at almost all radii in both geometries is higher than $0.01 (\frac{{ \text{\AA} }^{2}}{\text{ps}})$. That is the main reason for the long-range movement of C60.

A diffusion coefficient diagram for the C_60_ molecule at different radii and temperatures is presented in Fig. 9. The diffusion coefficient has been recognized as one of the essential characteristics in the motion of the C_60_. Comparing the diffusion coefficient diagram and obtained results from Figs. 5 and 6, the higher value for diffusion coefficient leads to more mobility for C_60_ molecules. The maximum distance has been taken related to the radius of $20 \; \text{\AA}$ at $400\; \text{K}$ in the concave substrate and the radius of $25 \; \text{\AA}$ at $600\; \text{K}$ in the cylindrical substrate, which corresponds to the highest diffusion coefficient in each geometry. Furthermore, according to the previous studies, for a diffusion coefficient higher than $0.01 (\frac{{ \text{\AA} }^{2}}{\text{ps}})$, the C_60_ molecule is able to move, but for smaller values, the molecule moves fluctuating or stationary^54^. Similarly, in the current study, in both geometry at almost all temperatures and radii, the diffusion coefficient is higher than $0.01 (\frac{{ \text{\AA} }^{2}}{\text{ps}})$ (Fig. 9), and the C_60_ molecule traveled a long-range distance properly. Therefore, the geometries studied in this research can be introduced as optimal geometries for C_60_ molecule. The types of C_60_ motion on different geometries were also summarized in Table S1.

### Nanocar

**Figure 10:**
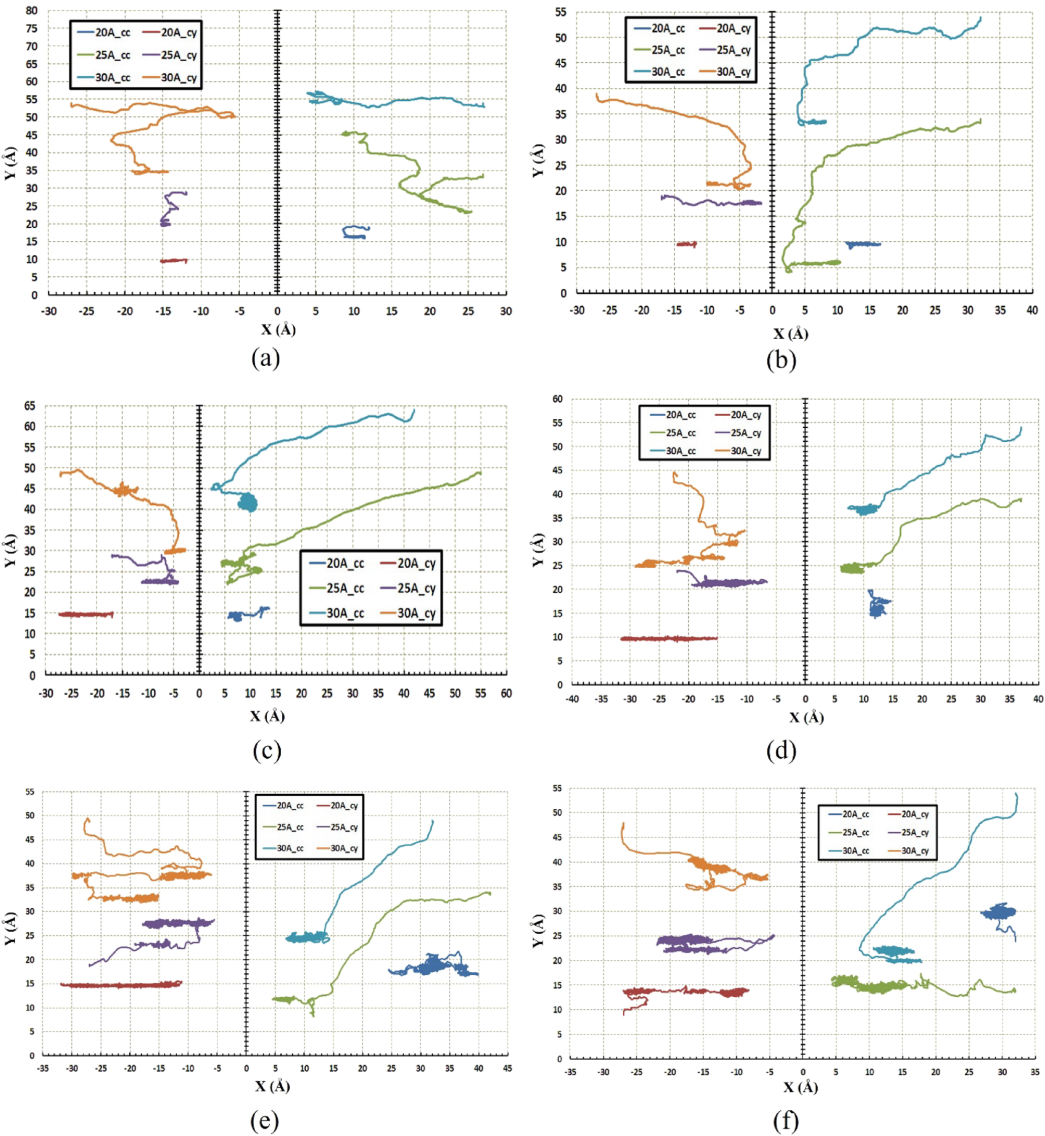
Trajectories of the nanocar on the concave (cc) and the cylindrical (cy) substrates during simulations (**a**) $75\; \text{K}$, (**b**) $150\; \text{K}$, (**c**) $300\; \text{K}$, (**d**) $400\; \text{K}$, (**e**) $500\; \text{K}$, and (**f**) $600\; \text{K}$. For the radius of $30 \; \text{\AA}$ at both geometries and the radius of $25 \; \text{\AA}$ at concave geometry, nanocar has long-range movement even at low temperatures like $75\; \text{K}$ and $150\; \text{K}$.

In the beginning, the regime of motion of the nanocar on the different geometries considering a range of temperature and radii is shown in Fig. 10. As mentioned in the analysis of the C_60_ molecule, it has demonstrated long-range movement even at low temperatures due to the high diffusion coefficient. Likewise, long-range movement of nanocar at low temperature can also be observed, especially at larger radii (Fig. 10a,b). Additionally, nanocar shows non-deflection motion in the radius of $20 \; \text{\AA}$ on the cylindrical substrate, which increases with the temperature significantly (Fig. 10).

**Figure 11:**
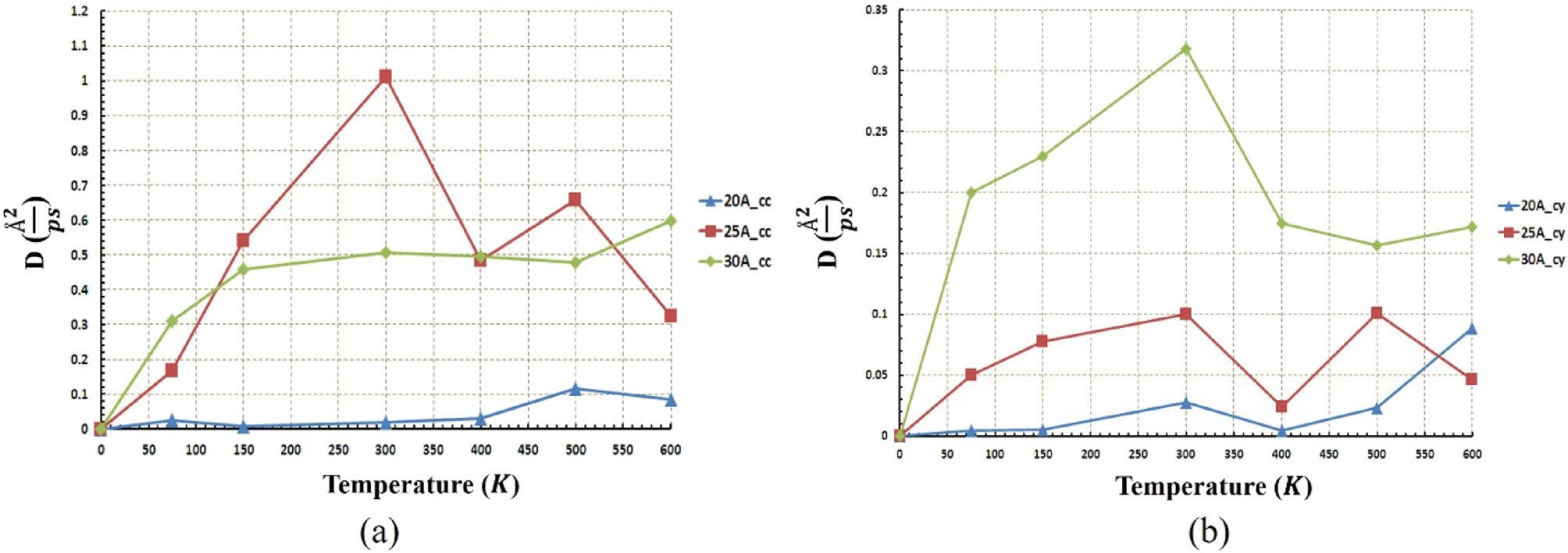
Diffusion coefficient of the nanocar at different temperatures and radii (**a**) for the concave (cc) substrates and (**b**) for the cylindrical (cy) substrates. The diffusion coefficient in concave geometry for almost all radii is higher than $0.01 (\frac{{ \text{\AA} }^{2}}{\text{ps}})$, but for cylindrical geometry only for the radius of $30 \; \text{\AA}$, the diffusion coefficient is higher than $0.01 (\frac{{ \text{\AA} }^{2}}{\text{ps}})$.

Figure 11a represents the diffusion coefficient of nanocar at different temperatures and different radii for the concave substrate. It can be observed that in the lower radius ($20 \; \text{\AA}$), the nanocar does not move properly so much such that it merely fluctuates along a certain length. This reciprocating movement of nanocar stems from the small amount of diffusion coefficient in the small radii. However, in the higher radii ($25 \; \text{\AA}$ and $30 \; \text{\AA}$), a long-range motion of nanocars has been observed due to the higher diffusion coefficient ($DC>0.01 (\frac{{ \text{\AA} }^{2}}{\text{ps}})$). In the large concave substrates ($25 \; \text{\AA}$ and $30 \; \text{\AA}$), the diffusion coefficient was greater than $0.01 (\frac{{\text{\AA} }^{2}}{\text{ps}})$ even at low temperatures (Fig. 11a). This characteristic makes them capable of being advantageous in quite a few objectives at low temperatures. In cylindrical geometry, the mentioned feature can be expressed only for radius $30 \; \text{\AA}$, according to Fig. 11b.

Moreover, nanocar deviates less in the cylindrical than the concave substrate in almost all temperatures and radii. This case mainly stems from the fact that the concave substrate has a higher surface-to-volume ratio than the cylindrical substrate, which absorb the nanocar to energetic points where the surface effects are much stronger. However, a longer distance has been taken by nanocar in the concave substrate in light of the nanocar flexible chassis. Actually, in the concave substrate, the chassis has more maneuvering space and only interacts with one surface, while in the cylindrical substrate, interaction occurs from both sides. Therefore, nanocar and its chassis demonstrate better movement in concave geometry.

**Figure 12:**
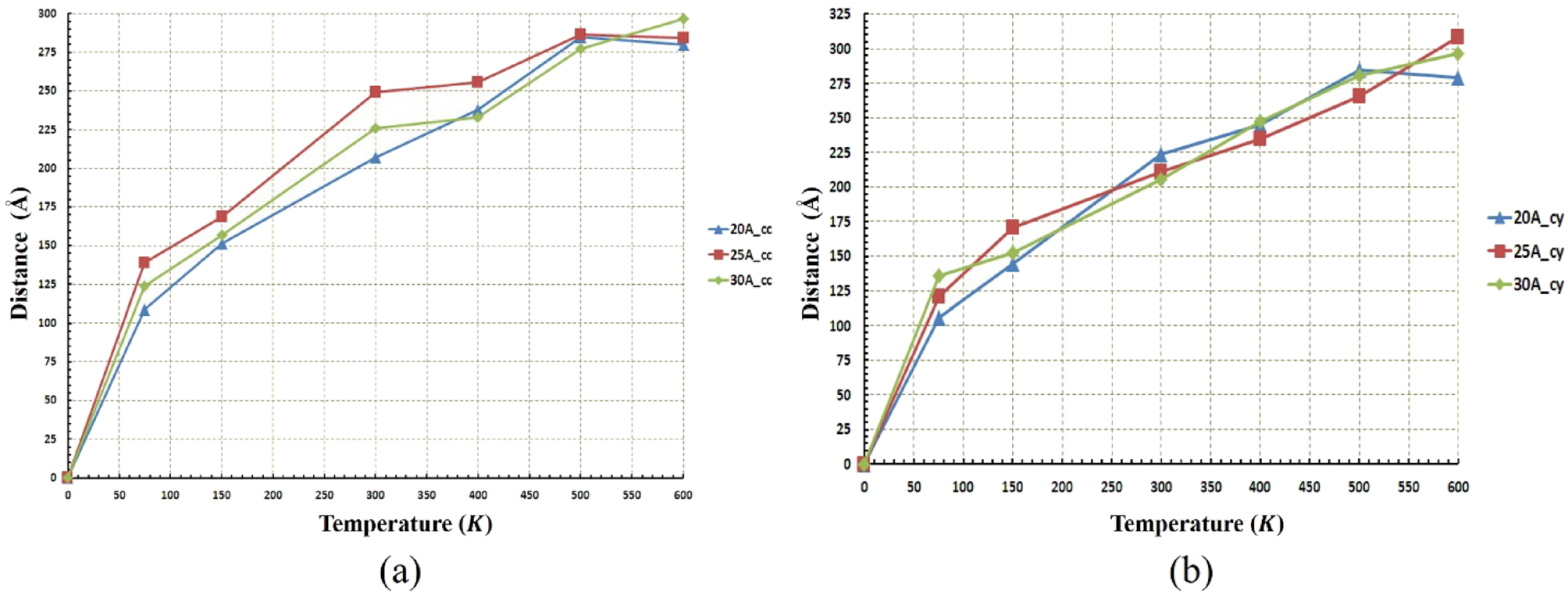
Distance traveled by the nanocar at different temperatures and radii (**a**) for the concave (cc) substrates and (**b**) for the cylindrical (cy) substrates. The shape of the geometry and the radius do not affect the nanocar's distance traveled. Therefore, at the same temperature, the distance traveled by nanocar in both geometries and any radius is identical.

**Figure 13:**
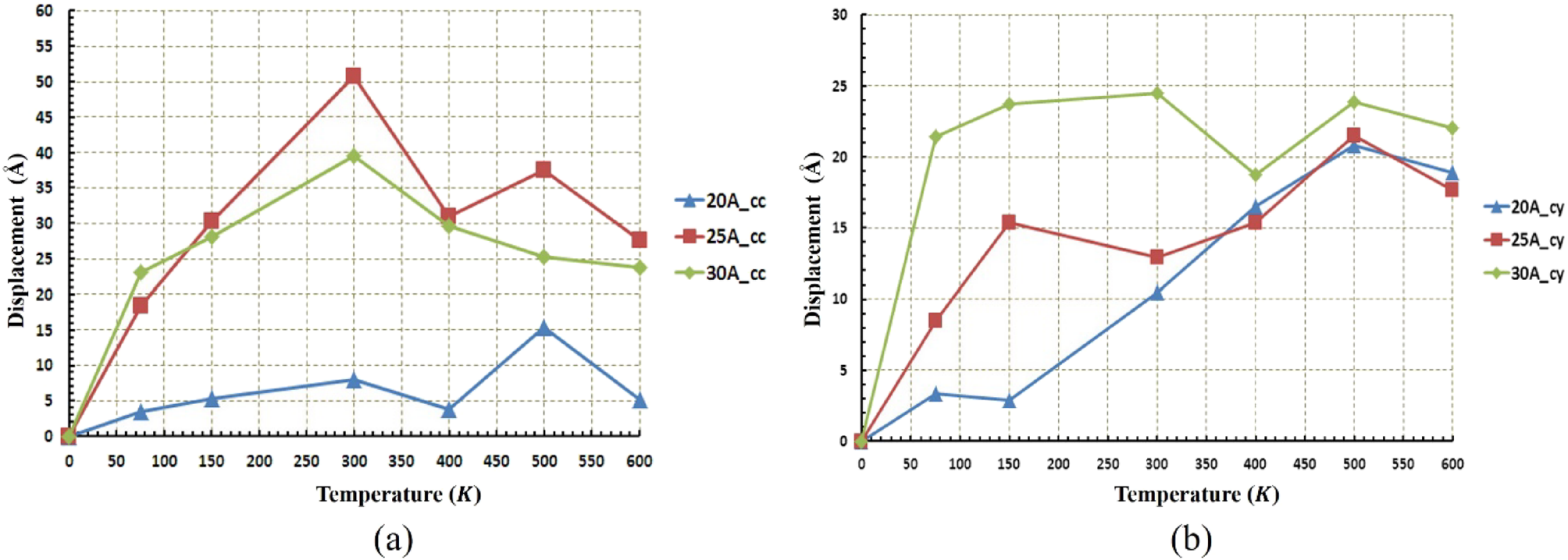
Displacement of the nanocar at different temperatures and radii (**a**) for the concave (cc) substrates (**b**) for the cylindrical (cy) substrates. The average displacement in concave geometry is higher than the cylindrical geometry. The maximum displacement of nanocar has occurred in the radius of $25 \; \text{\AA}$ concave substrates and at $300\; \text{K}$.

As mentioned before, the regime of motion of nanocar is not only temperature-dependent, but affected by radius changes as well. This issue and its relationship with the diffusion coefficient were revealed in Figs. 10 and 11. In the next stage, for more precise investigation, the movement of nanocar in different conditions is shown in Figs. 12 and 13, representing the path length and the displacement diagrams of nanocar, respectively. For better undressing about nanocar’s motion, the average velocity of nanocar has been demonstrated in Fig. S1.

The equal distances have been traveled by nanocar in both substrates considering the same radii and temperatures (Fig. 12). In both geometries and for all radii, with increasing temperature, an ascending trend was observed for the distance traveled by nanocar. However, in both geometries, the effect of the radius is clearly significant, so much such that it cannot be demonstrated that increasing or decreasing the value of radius at different temperatures leads to the same trend for distance traveled by nanocar.

As mentioned before, for a radius of $20 \; \text{\AA}$ in concave geometry, the value of the diffusion coefficient of the nanocar is less than $0.01 (\frac{{ \text{\AA} }^{2}}{\text{ps}})$ (Fig. 11a), which results in fluctuating motion of the nanocar in these conditions. By considering Figs. 12 and 13 simultaneously, a better understanding of the deviations and fluctuations above can be obtained at different conditions for the nanocar. With increasing radius in concave geometry, the diffusion coefficient increased significantly (Fig. 11a), and consequently, long-range movements of nanocar have been observed, specified in Fig. 13a. In concave geometry, maximum displacement was denoted to the radios of $25 \; \text{\AA}$, in which the greatest displacement was observed at the $300\; \text{K}$ (Fig. 13a). Important to note this maximum displacement corresponds to the highest diffusion coefficient of nanocar in concave geometry (Fig. 11a).

In cylindrical geometry, at low temperatures in the radius of $20 \; \text{\AA}$, the fluctuating motion of nanocar occurred, and no suitable displacement was observed. In addition, increasing the temperature leads to a higher diffusion coefficient (Fig. 11b), which provides a long-range displacement for nanocar (Fig. 13b).

In the other two radii of this geometry ($25 \; \text{\AA}$ and $30 \; \text{\AA}$), long-range displacements were observed from the beginning (Fig. 13b). By increasing the radius, the amount of displacement has been increased at different temperatures so that in the radius of $30 \; \text{\AA}$, the nanocar has the highest displacement at all temperatures (Fig. 13b). In this radius, the maximum displacement was observed at $300\; \text{K}$, corresponding to the highest nanocar diffusion coefficient in cylindrical geometry (Figs. 11b and 13b). The types of C_60_ motion on different geometries were also summarized in Table S1. The types of nanocar motion on different geometries were also summarized in Table S2.

### Nanotruck

**Figure 14:**
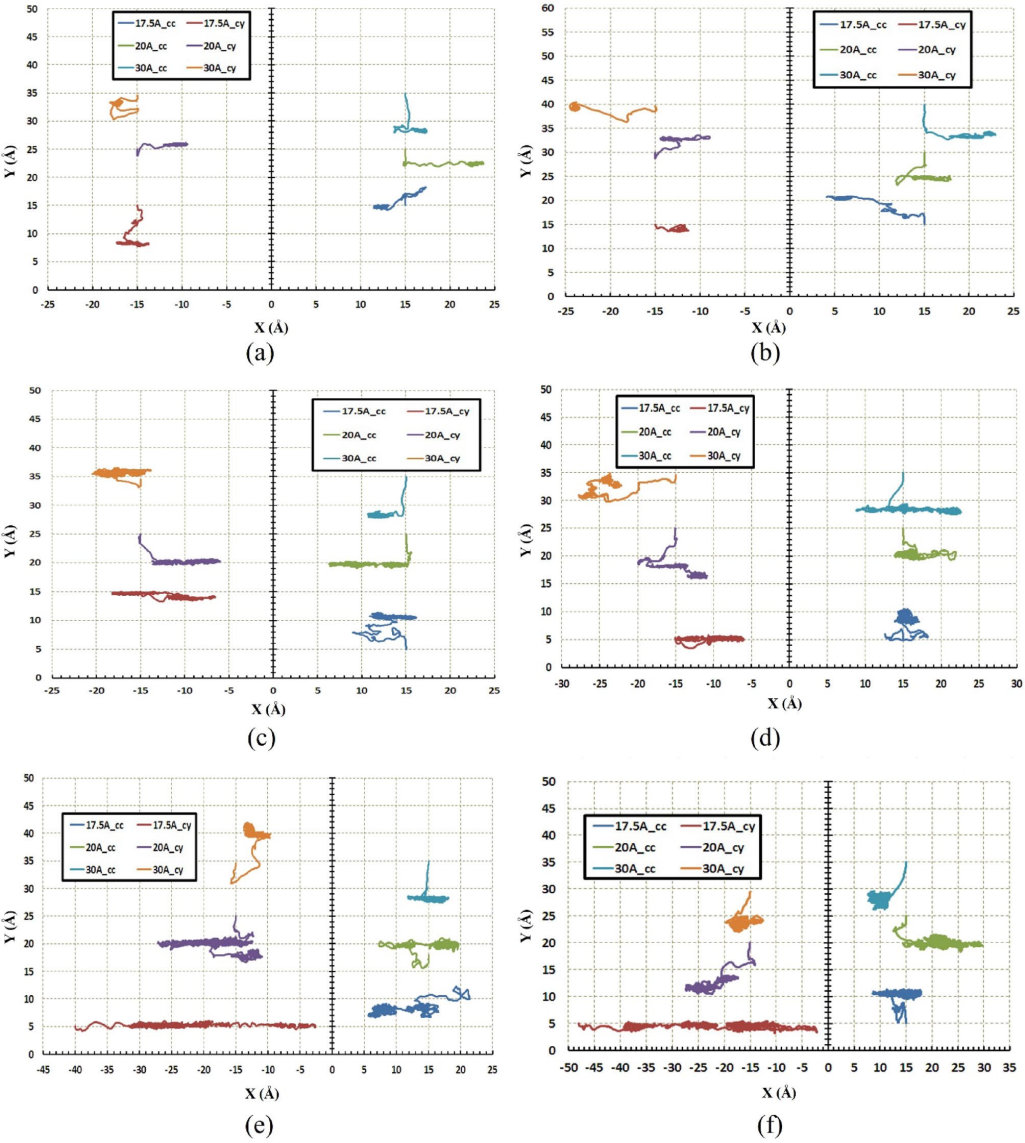
Trajectories of the nanotruck on the concave (cc) and the cylindrical (cy) substrates during simulations (**a**) $75\; \text{K}$, (**b**) $150\; \text{K}$, (**c**) $300\; \text{K}$, (**d**) $400\; \text{K}$, (**e**) $500\; \text{K}$, and (**f**) $600\; \text{K}$. Nanotruck has fluctuated at almost all temperatures in both geometries except the radius of $17.5 \; \text{\AA}$ cylindrical substrates at $500\; \text{K}$ and $600\; \text{K}$ that nanotruck has long-range movement.

Figure 14 represents graphs of nanotruck movement on the cylindrical and concave substrates. Unlike C_60_ and nanocar, which display long-range displacements, a fluctuating and indirect motion has been observed for nanotruck on both geometries at almost all temperatures.

Nanotruck fluctuation is not observed only in the radius of $17.5 \; \text{\AA}$ at temperatures of $500\; \text{K}$ and $600\; \text{K}$ in cylindrical geometry (Fig. 14e,f). In this radius, the nanotruck shows a long-range displacement as well as direct movement.

It was expected in the concave geometry, similar to nanocar, nanotruck absorb to the high-energy edge points where the surface effects are much stronger, but nanotruck shows reciprocating motion. It seems that the nanotruck energy was not sufficient to overcome this fluctuating motion. As a result, the absorption toward high-energy points does not happen unless the nanotruck goes out of its fluctuating motion.

**Figure 15:**
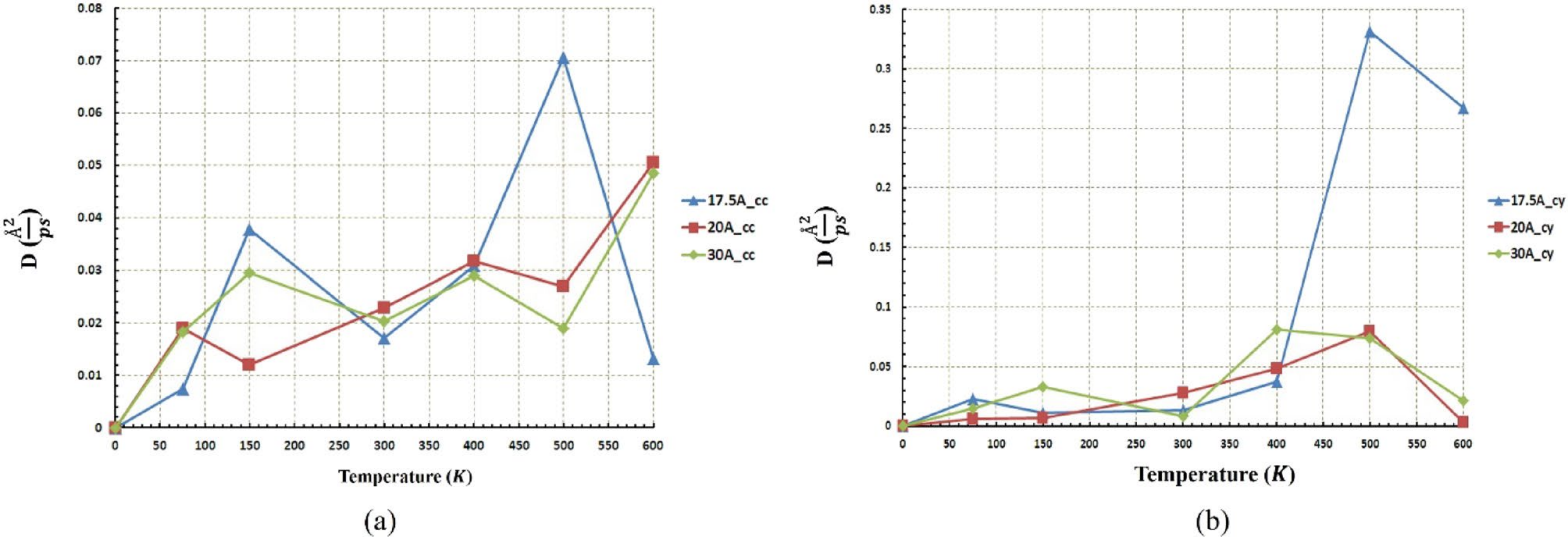
Diffusion coefficient of the nanotruck at different temperatures and radii (**a**) for the concave (cc) substrates and (**b**) for the cylindrical (cy) substrates. Naotruck’s diffusion coefficient in concave geometry for all radii is lower than $0.01 (\frac{{ \text{\AA} }^{2}}{\text{ps}})$. In addition, nanotruck’s diffusion coefficient is lower than $0.01 (\frac{{ \text{\AA} }^{2}}{\text{ps}})$ in all radii except the radius of $17.5 \; \text{\AA}$ at $500\; \text{K}$ and $600\; \text{K}$ in cylindrical geometry.

To investigate the nanotruck motion more accurately and better analyze its behavior, diffusion coefficient diagrams of nanotruck for both geometries were illustrated in Fig. 15. nanotruck diffusion coefficient in concave geometry is located at the boundary between fluctuating and long-range motion (previously mentioned as $0.01 (\frac{{\text{\AA} }^{2}}{\text{ps}})$). This is the main reason for the fluctuating motion of nanotruck at all temperatures and radii except the radius of 17.5 Å at $500\; \text{K}$, which is not significant compared to nanocar travel ranges (Fig. 15a). In cylindrical geometry, except in the radius of $17.5 \; \text{\AA}$ at $500\; \text{K}$ and $600\; \text{K}$, the diffusion coefficient of the nanotruck remains in the fluctuation range (Fig. 15b). In addition, at high temperatures in the radius of $17.5 \; \text{\AA}$, the diffusion coefficient of the nanotruck is sufficiently large so that nanotruck could overcome its fluctuating movement (Red lines in Fig. 14e,f).

**Figure 16:**
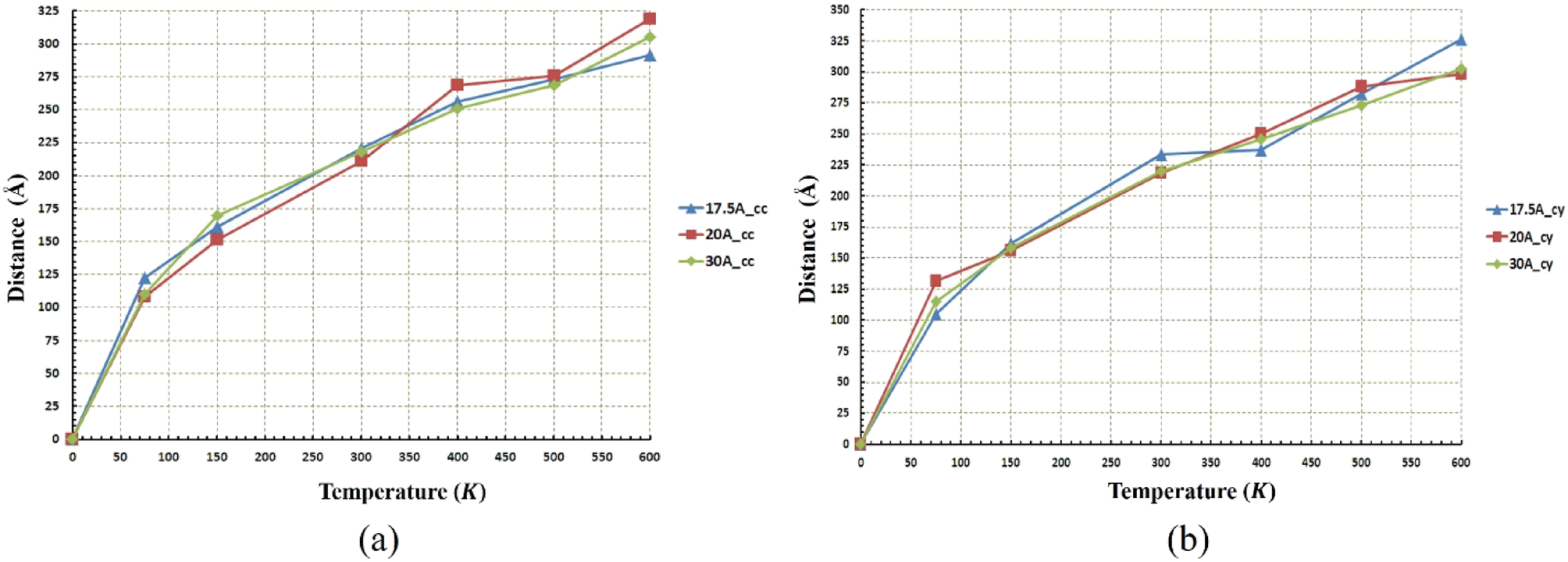
Distance traveled by the nanotruck at different temperatures and radii (**a**) for the concave (cc) substrates and (**b**) for the cylindrical (cy) substrates. The shape of the geometry and the radius do not affect the nanotruck's distance traveled. Hence, at the same temperature, the distance traveled by nanotruck in both geometries and any radius is identical.

**Figure 17:**
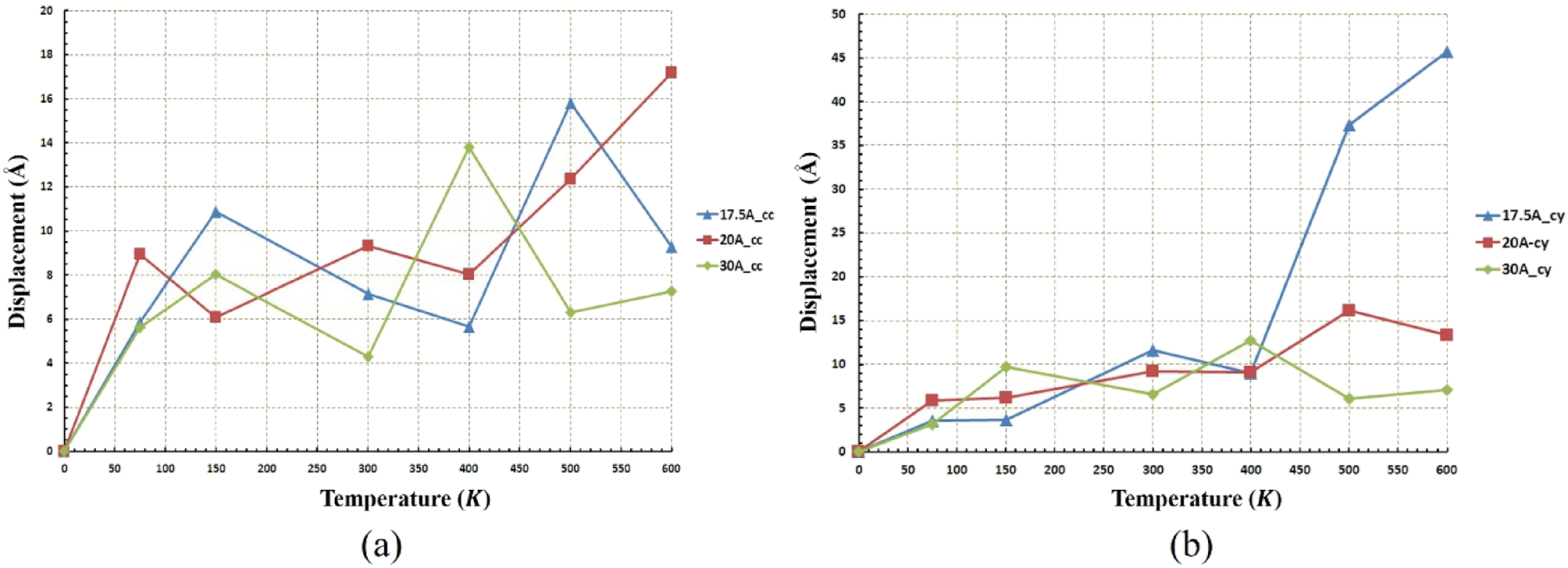
Displacement of the nanotruck at different temperatures and radii (**a**) for the concave (cc) substrates and (**b**) for the cylindrical (cy) substrates. Due to fluctuating movement of nanotruck in almost all conditions, the average displacement in both geometries is the same. The maximum displacement of nanotruck has occurred in the radius of $17.5 \; \text{\AA}$ cylindrical substrates at $600\; \text{K}$.

Following the process performed in the analysis of nanotruck, Figs. 16 and 17 represent the distance traveled and displacement diagrams for nanotruck, respectively. The average velocity of nanotruck has been demonstrated Fig. S2 to attain better understanding about nanotruck’s motion.

Comparing the two diagrams presented in Fig. 16, the distance traveled by the nanotruck is approximately equal at the same temperature and radius in both geometries. Unlike the temperature, by radius changes, no specific trend has been achieved since the impact of radius in different geometries is influenced by temperature changes.

The same distance has been traveled almost in all radii at $600\; \text{K}$ and $500\; \text{K}$ (Fig. 16b), but as mentioned earlier, due to the low diffusion coefficient in the $20 \; \text{\AA}$ and $30 \; \text{\AA}$ radii, the nanotruck motion remained in fluctuating range (Fig. 17b). In these two radii, despite the large distances traveled by nanotruck, short displacements have been performed by nanotruck. However, a long-range displacement was observed in the radius of $17.5 \; \text{\AA}$, which corresponds to the highest diffusion coefficient of nanotracks in this geometry (Fig. 17b).

Figure 17a confirms the previously presented analyzes of the fluctuating motion of nanotruck in the concave geometry. The maximum displacement of the nanotruck in concave geometry was observed at $17.5 \; \text{\AA}$, which occurred at a temperature of $600\; \text{K}$. It is clear that the nanotruck did not experience an excellent long-range motion in any of the simulation conditions in concave geometry in the light of the low diffusion coefficient (Figs. 15a and 17a). All in all, nanotruck, unlike C_60_ and nanocar, has not revealed acceptable performance in most conditions, except on the $17.5 \; \text{\AA}$ cylindrical substrate at high temperatures. The types of nanotruck motion on different geometries were also summarized in Table S3.

## Conclusion

Simulation can be a valuable tool for investigating physical/chemical phenomena^26,50–59^. In the current project, the motion of C_60_, nanocar, and nanotruck on the cylindrical and concave substrates, have been investigated. Considering the presented results, the deviations of C_60_, nanocar, and nanotruck were significantly undersized compared to the previous studies^31^. This issue stems from the fact that the substrate's geometries selected in this study, restricted the non-desired directions for nano-machines. Besides, the substrates' curved shape provides a better interaction between the gold surface and C_60_ and nano-machines' wheels. The C_60_ and nanocar indicated less deviation on the cylindrical substrate than concave geometry due to fewer surface effects. In another explanation, this surface effect attracts the C_60_ and nanocar into the high-energy area (edges); thus, molecules have experienced more deviation on the concave substrate. However, since nanotruck motion was located in a small fluctuating range, the nanotruck did not absorb the high-energy area (edges). As far as potential energy is concerned, the mean and maximum potential energies were not affected by temperature changes. Therefore, this parameter is not a cause for less mobility of C_60_ in the aforementioned items. In addition, the average and maximum potential energy were increased in the cylindrical substrate; it is twice as concave, with increasing radius in both geometries. Additionally, equal distances have been traveled by nanocar and nanotruck on both substrates considering the same radii and temperatures. In both geometries and for all radii, with increasing the temperature, an ascending trend was observed for the distance traveled by nanocar. However, in both geometries, the effect of the radius is clearly significant, so much such that it cannot be demonstrated that increasing or decreasing the value of radius at different temperatures, leads to the same trend for distance traveled by nanocar. As previously mentioned, the diffusion coefficient is recognized as the leading characteristic for defining the nano-machines' motion, which is predominantly affected by the temperature. This study revealed that the diffusion coefficient is similarly influenced notably by substrates' geometry (radius). Nonetheless, changing the radius did not specify a particular trend for the diffusion coefficient. As a result, considering the obtained radii for this study, long-range motion can be reached for nano-machines even at low temperatures ($75\; \text{K}$ or $150\; \text{K}$). The motion of the nanotruck on the smallest cylindrical geometry was acceptably in a direct path at high temperatures. As a result, using nanotruck in the applications, which were carried out at high temperatures ($500\; \text{K}$ to $600\; \text{K}$), is strongly recommended. In addition, employing the nanocar on all cylindrical substrates stated in this study is suggested extensively due to its sufficient diffusion coefficient  $\left (DC<< 0.01 \left(\frac{{\text{\AA} }^{2}}{\text{ps}} \right) \right)$.

## Supplementary Information

Supplementary Information.

## Data Availability

The data of this study is available upon reasonable request from the corresponding author.

## Abbreviations

RBMD: Rigid-body molecular dynamics
STM: Scanning tunneling microscopy
EAM: Embedded atom method
MM: Molecular mechanics
FCC: Face-centered cubic
RHF: Restricted Hartree–Fock
LAMMPS: Large scale atomic/molecular massively parallel simulator
VMD: Visual molecular dynamics
