# Supplementary material for: Investigation of the motion of fullerene-wheeled nano-machines on thermally activated curved gold substrates
Source: Sci Rep. 2022 Oct 29;12:18255. doi: 10.1038/s41598-022-22517-1 (PMC9617915; doi:10.1038/s41598-022-22517-1)
Supplement: Supplementary file 1 — Supplementary Information. [file 41598_2022_22517_MOESM1_ESM.docx]

# Supporting Information for

### Investigation of the motion of fullerene-wheeled nano-machines on thermally activated curved gold substrates

### Mohammad Ali Bakhtiari ^a^, Saeed Seifi ^a^, Mahdi Tohidloo ^a^, Amir Shamloo ^a, *^

**^a^ School of Mechanical Engineering, Sharif University of Technology, Tehran, Iran**

# ^*^Corresponding Author: Dr. A. Shamloo, School of Mechanical Engineering, Sharif University of Technology, Azadi Ave., Tehran, IRAN, Tel: 98-21-66165691, Fax: 98-21-66165599, email: [shamloo@sharif.edu](mailto:shamloo@sharif.edu)

# Table of content

# Supplementary Note ……………………………………………………………………...……S3

**Figure S1.** Average velocity of nanocar. S6

[**Figure S2.** Average velocity of nanotruck S7](#_Toc47522915)

**Table S1.** Types of $C_{60}$ motion S8

[**Table S2.** Types of nanocar motion S9](#_Toc47522919)

[**Table S3.** Types of nanotruck motion S10](#_Toc47522917)

# Supplementary Notes

**Supplementary Note 1-** **Types of** $\boldsymbol{C}_{\boldsymbol{60}}$ **motion in different situations**

**Table S1.** has reported the types of $C_{60}$ motion under different conditions. Based on **Table S1**, $10.4$ percent of movements were short-range, which almost occurred at low temperatures like $75 K$ and $150 K$. $C_{60}$ molecule for $27.1$ percent of situations has fluctuated; Hence, $C_{60}$ showed a long-range movement for $62.5$ percent of conditions. Therefore, it can be noted that the proposed geometries in this study, are optimized for $C_{60}$ molecule.

**Supplementary Note 2- Types of nanocar motion in different situations**

**Table S2.** has presented the nanocar’s motion under different conditions. For $69.4$ percent of motions, the long-range movement has occurred so much such the long-range movement was happened even at at low temperatures that correspond to the radius effect on nanocar’s motion that did not consider in previous studies. Besides, nanocar displayed short-range and fluctuation motion in $11.1$ percent and $19.5$percent of conditions, respectively. Short-range and fluctuation movement almost have occurred at low temperatures like $75 K$ and $150 K$.

**Supplementary Note 3*-* Types of nanotruck motion in different situations**

Based on **Table S3**, nanotruck for almost $83$ percent of conditions had short-range or fluctuated motions. So that, $19.5$ percent and $63.5$ percent of nanotruck motions in different situations have been short-range and fluctuated, respectively. At higher temperatures like $500 K$ and $600 K$, the nanotruck demonstrated a long-range movement, but it is only less than $17$ percent of situations.

**Supplementary Note 4*-* Average velocity of nanocar**

The mean velocity in the concave geometry is higher than the cylindrical geometry Due to the existence of energetic points (the points with a high surface-to-volume ratio) in the concave geometry; Nanocar is adsorbed to these points, which makes nanocar’s movement faster. In addition, **Figure S1** represents the radius effect on the nanocar's motion. The radius effect does not clear, So that the nanocar motion may be different at any temperature.

**Supplementary Note 5*-* Average velocity of nanotruck**

Corresponding to the nanocar flexible chassis, the affection of the radius and the energetic points on the nanotruck is less than nanocar. Hence, the velocity of the nanotruck is lower than the nanocar in all conditions. similarly, in the case of the nanotruck, the effect of the radius does not clear. So that the radius effect at any temperature may be different. Based on **Figure S2**, it could be concluded that the concave and cylindrical substrate is an appropriate substrates for nanotruck at low temperatures and high temperatures, respectively.

# Figure captions

***Figure S1.*** *Average velocity of nanocar at different temperatures on a) concave (CC), and b) cylindrical (CY) substrate.*

**Figure S2.** Average velocity of nanotruck at different temperatures on a) concave (CC), and b) cylindrical (CY) substrate.

**Figure S1**


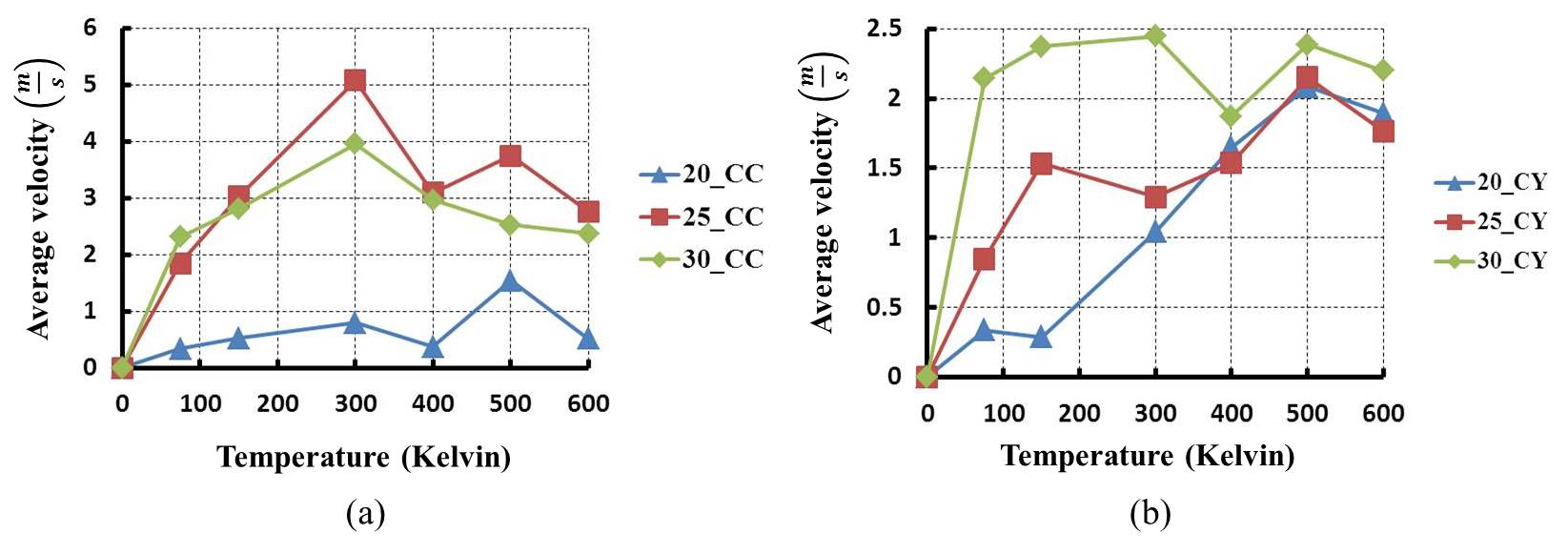


**Figure S2**


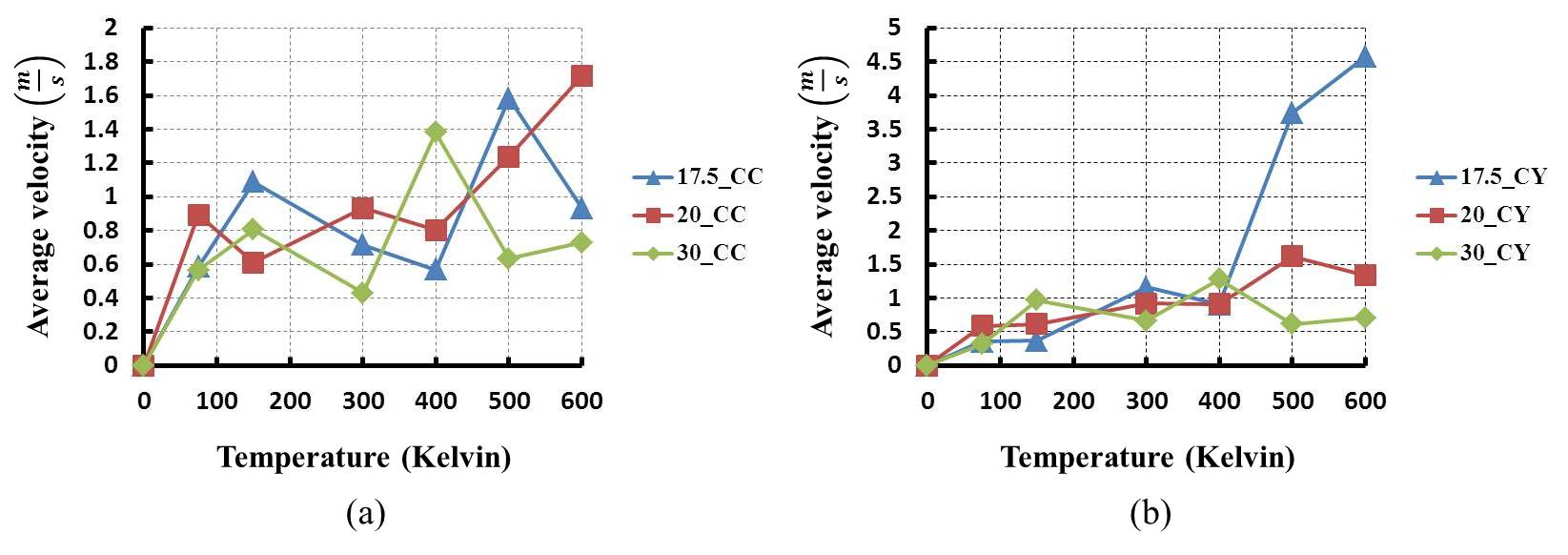


**Table S1.** Types of $C_{60}$ motion in different situations

| Temperature  Radius | $\boldsymbol{75}\boldsymbol{K}$ | $\boldsymbol{150}\boldsymbol{K}$ | $\boldsymbol{300}\boldsymbol{K}$ | $\boldsymbol{400}\boldsymbol{K}$ | $\boldsymbol{500}\boldsymbol{K}$ | $\boldsymbol{600}\boldsymbol{K}$ |
| --- | --- | --- | --- | --- | --- | --- |
| $\boldsymbol{17.5Å}$  (Concave) | Short-range | Fluctuation | Fluctuation | Long-range | Long-range | Long-range |
| $\boldsymbol{20Å}$  (Concave) | Short-range | Fluctuation | Long-range | Long-range | Long-range | Long-range |
| $\boldsymbol{25Å}$  (Concave) | Fluctuation | Fluctuation | Fluctuation | Long-range | Long-range | Long-range |
| $\boldsymbol{30Å}$  (Concave) | Fluctuation | Long-range | Long-range | Long-range | Long-range | Long-range |
| $\boldsymbol{17.5Å}$  (Cylinder) | Short-range | Short-range | Long-range | Fluctuation | Long-range | Fluctuation |
| $\boldsymbol{20Å}$  (Cylinder) | Long-range | Fluctuation | Long-range | Long-range | Long-range | Long-range |
| $\boldsymbol{25Å}$  (Cylinder) | Short-range | Long-range | Fluctuation | Long-range | Long-range | Long-range |
| $\boldsymbol{30Å}$  (Cylinder) | Fluctuation | Fluctuation | Long-range | Long-range | Long-range | Long-range |

**Table S2.** Types of nanocar motion in different situations

| Temperature  Radius | $\boldsymbol{75}\boldsymbol{K}$ | $\boldsymbol{150}\boldsymbol{K}$ | $\boldsymbol{300}\boldsymbol{K}$ | $\boldsymbol{400}\boldsymbol{K}$ | $\boldsymbol{500}\boldsymbol{K}$ | $\boldsymbol{600}\boldsymbol{K}$ |
| --- | --- | --- | --- | --- | --- | --- |
| $\boldsymbol{20Å}$  (Concave) | Short-range | Short-range | Fluctuation | Long-range | Long-range | Long-range |
| $\boldsymbol{25Å}$  (Concave) | Long-range | Long-range | Long-range | Long-range | Long-range | Long-range |
| $\boldsymbol{30Å}$  (Concave) | Long-range | Long-range | Long-range | Long-range | Long-range | Long-range |
| $\boldsymbol{20Å}$  (Cylinder) | Short-range | Short-range | Fluctuation | Fluctuation | Long-range | Fluctuation |
| $\boldsymbol{25Å}$  (Cylinder) | Fluctuation | Fluctuation | Fluctuation | Long-range | Long-range | Long-range |
| $\boldsymbol{30Å}$  (Cylinder) | Long-range | Long-range | Long-range | Long-range | Long-range | Long-range |

**Table S3.** Types of nanotruck motion in different situations

| Temperature  Radius | $\boldsymbol{75}\boldsymbol{K}$ | $\boldsymbol{150}\boldsymbol{K}$ | $\boldsymbol{300}\boldsymbol{K}$ | $\boldsymbol{400}\boldsymbol{K}$ | $\boldsymbol{500}\boldsymbol{K}$ | $\boldsymbol{600}\boldsymbol{K}$ |
| --- | --- | --- | --- | --- | --- | --- |
| $\boldsymbol{17.5Å}$  (Concave) | Fluctuation | Fluctuation | Fluctuation | Short-range | Long-range | Fluctuation |
| $\boldsymbol{20Å}$  (Concave) | Fluctuation | Fluctuation | Fluctuation | Fluctuation | Long-range | Fluctuation |
| $\boldsymbol{30Å}$  (Concave) | Short-range | Fluctuation | Fluctuation | Fluctuation | Fluctuation | Short-range |
| $\boldsymbol{17.5Å}$  (Cylinder) | Short-range | Short-range | Fluctuation | Fluctuation | Long-range | Long-range |
| $\boldsymbol{20Å}$  (Cylinder) | Fluctuation | Fluctuation | Fluctuation | Fluctuation | Long-range | Long-range |
| $\boldsymbol{30Å}$  (Cylinder) | Short-range | Fluctuation | Fluctuation | Fluctuation | Fluctuation | Short-range |
